# Supplementary material for: Evolution of Highly Biocompatible and Thermally Stable YVO4:Er3+/Yb3+ Upconversion Mesoporous Hollow Nanospheriods as Drug Carriers for Therapeutic Applications
Source: Nanomaterials (Basel). 2022 Jul 22;12(15):2520. doi: 10.3390/nano12152520 (PMC9332312; doi:10.3390/nano12152520)
Supplement: Supplementary file 1 [file nanomaterials-12-02520-s001.zip › nanomaterials-1805804-supplementary.pdf]

Supplementary Material

Evolution of Highly Biocompatible and Thermally Stable YVO_4:Er^{3+}/Yb^{3+} Upconversion Mesoporous Hollow Nanospheriods as Drug Carriers for Therapeutic Applications

Eluri Pavitra [1], Hoomin Lee [1], Seung Kyu Hwang [1], Jin Young Park [2], Young-Kyu Han [3], Ganji Seeta Rama Raju [3,*] and Yun Suk Huh [1,*]

[1] Department of Biological Engineering, Biohybrid Systems Research Center (BSRC), Inha University, Incheon 22212, Korea; pavitra@inha.ac.kr (E.P.); hmlee8907@inha.ac.kr (H.L.); 22152213@inha.edu (S.K.H.)
[2] Department of Electrical, Electronics and Software Engineering, Pukyong National University, Yongdang Campus, Busan 48547, Korea; pjy0329@pknu.ac.kr
[3] Department of Energy and Materials Engineering, Dongguk University-Seoul, Seoul 04620, Korea; ykenergy@dongguk.edu
* Correspondence: gseetaramaraju7@dongguk.edu or gseetaramaraju@live.in (G.S.R.R.); yunsuk.huh@inha.ac.kr (Y.S.H.)

Characterizations:

The morphological studies were carried using field-emission scanning electron microscopy (FE-SEM) (Hitachi-SU8010, Japan) and the field-emission transmission electron microscopy (FE-TEM: JEM-2100F/JEOL) measurements. The X-ray diffraction (XRD) patterns of YC:Er^{3+}/Yb^{3+} NSs and YVO_4:Er^{3+}/Yb^{3+} UC-MHNSPs were measured using X'Pert Pro MRD system (PANalytical) with ceramic Cu target (CuKα= 1.5406 Å), X-ray generator of 3kW 60kV/60mA, and scan speed of 1 deg/sec. The elemental composition was tested using energy dispersive X-ray spectrometer attached to FE-SEM instrument (PANalytical Holland). Nitrogen adsorption/desorption analysis of UC-MHNSPs were measured using Specific Surface Analyzer (3Flex) system. The UC emission spectra were recorded using a 980 nm continuous wave laser diode (1W) as the pumping source (Model No.: PSU-III-LED, Input: 85~264VAC 47~63Hz 2A, Changchun new industries, Optoelectronics Tech. Co. LTD) and detected by Ocean Optics software. The Zeta potential of YVO_4:Er^{3+}/Yb^{3+} UC-MHNSPs were measured by Zeta Potential Analyzer (ELS-Z) system with voltage current range of 0 to 50 mA. For Zeta potential measurement, flow cell was used with water as diluent. The in vitro imaging was captured using an inverted fluorescence microscope (Olympus CKX53).

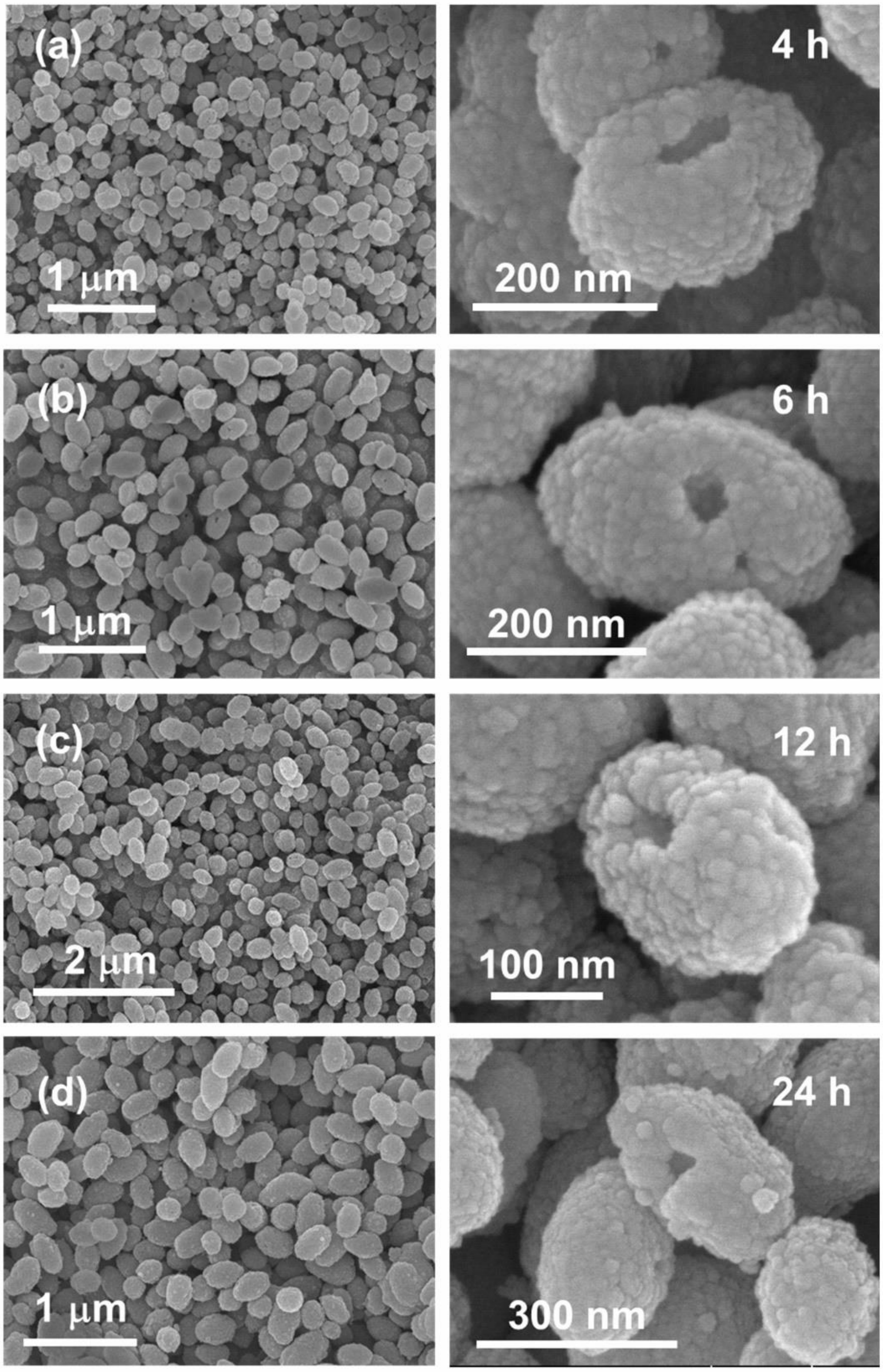

Figure S1. FE-SEM images of YVO_4:Er^{3+}/Yb^{3+} UC-MHNSPs at different reaction timings of (**a**) 4 h, (**b**) 6 h, (**c**) 12 h, (**d**) 24 h respectively.

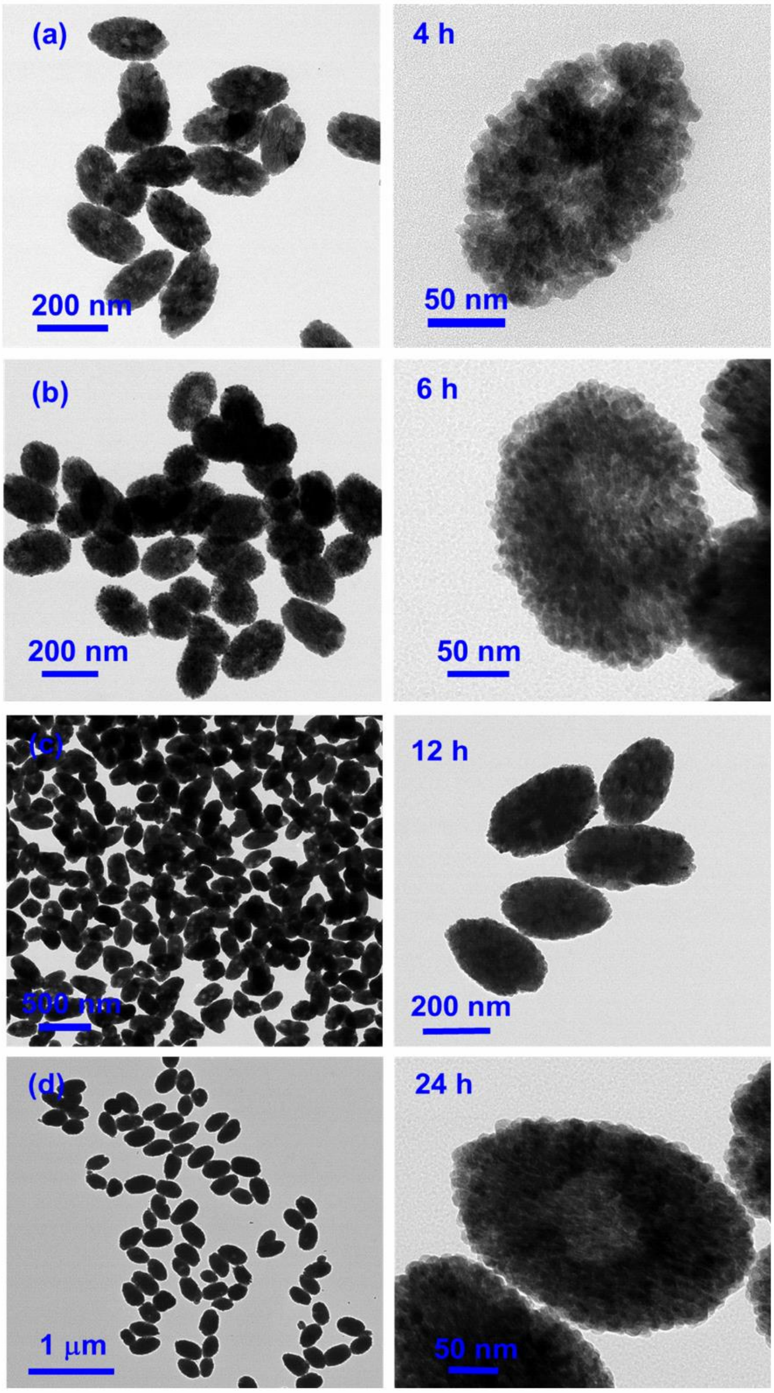

Figure S2. FE- TEM images of YVO_4:Er^{3+}/Yb^{3+} UC-MHNSPs at different reaction timings of (**a**) 4 h, (**b**) 6 h, (**c**) 12 h, (**d**) 24 h respectively.

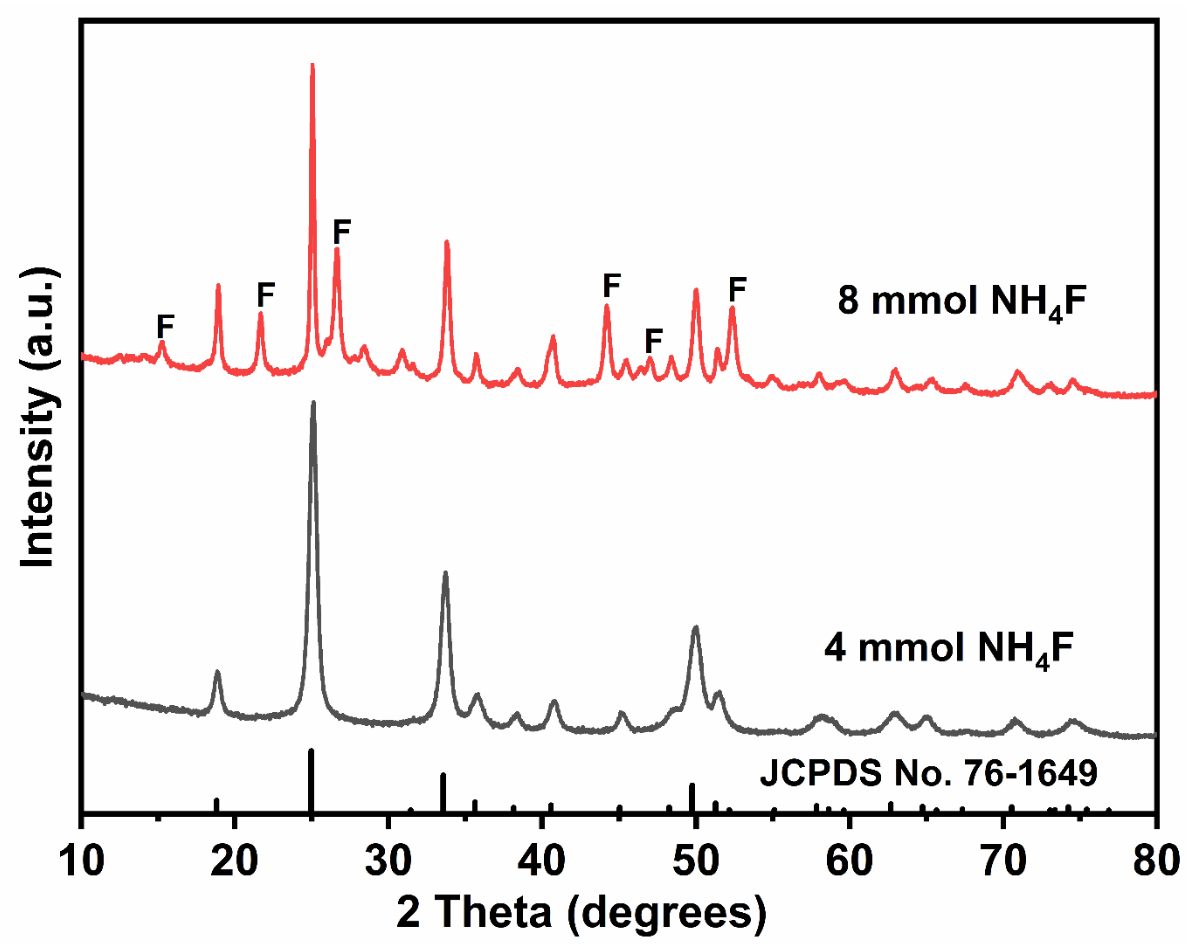

Figure S3. XRD patterns of $YVO_4:Er^{3+}/Yb^{3+}$ UC-MHNSPs with two different concentrations of NH_4F flux.

Table S1. Atomic parameters, occupancy, and atomic displacement parameters (U_{iso} [$Å^2$]) of $YVO_4:Er^{3+}/Yb^{3+}$ UC-MHNSPs. .

Atoms	Wyckoff	x/a	y/b	z/c	Occupancy	U[Å2]
Y	8 c	0.00000	0.75000	0.12500	0.8900	0.00883
Er	8 c	0.00000	0.75000	0.12500	0.0100	0.03343
Yb	8 c	0.00000	0.75000	0.12500	0.1000	0.49316
V	16 h	0.00000	0.25000	0.37500	1.0000	0.02598
O	16 h	0.00000	0.073426	0.192788	1.0000	0.00438
